# Non-Mendelian transmission of accessory chromosomes in fungi

**DOI:** 10.1007/s10577-022-09691-8

**Published:** 2022-07-26

**Authors:** Jovan Komluski, Eva H. Stukenbrock, Michael Habig

**Affiliations:** 1 Environmental Genomics, Christian-Albrechts University of Kiel, Kiel, Germany; 2 Max Planck Institute for Evolutionary Biology, Plön, Germany

**Keywords:** Non-Mendelian transmission, Accessory chromosomes, Meiotic drive, Disomy, Fungi

## Abstract

Non-Mendelian transmission has been reported for various genetic elements, ranging from small transposons to entire chromosomes. One prime example of such a transmission pattern are B chromosomes in plants and animals. Accessory chromosomes in fungi are similar to B chromosomes in showing presence/absence polymorphism and being non-essential. How these chromosomes are transmitted during meiosis is however poorly understood—despite their often high impact on the fitness of the host. For several fungal organisms, a non-Mendelian transmission or a mechanistically unique meiotic drive of accessory chromosomes have been reported. In this review, we provide an overview of the possible mechanisms that can cause the non-Mendelian transmission or meiotic drives of fungal accessory chromosomes. We compare processes responsible for the non-Mendelian transmission of accessory chromosomes for different fungal eukaryotes and discuss the structural traits of fungal accessory chromosomes affecting their meiotic transmission. We conclude that research on fungal accessory chromosomes, due to their small size, ease of sequencing, and epigenetic profiling, can complement the study of B chromosomes in deciphering factors that influence and regulate the non-Mendelian transmission of entire chromosomes.

## Introduction

Non-Mendelian transmission represents a deviation from Mendel’s axiom of equal probabilities for a heterozygous gene or chromosome to be transmitted to the meiotic progeny. Therefore, any process that increases or decreases the probability of transmission of a genetic element during meiosis will result in such non-Mendelian transmission. Non-Mendelian transmission is widespread and can affect short sequences as well as entire chromosomes (Hurst and Werren 2001). Here, we will focus on non-Mendelian segregation affecting entire chromosomes in fungi—which is restricted to mainly two processes: (i) meiotic chromosome drives and (ii) losses and disomies of entire chromosomes during meiosis.
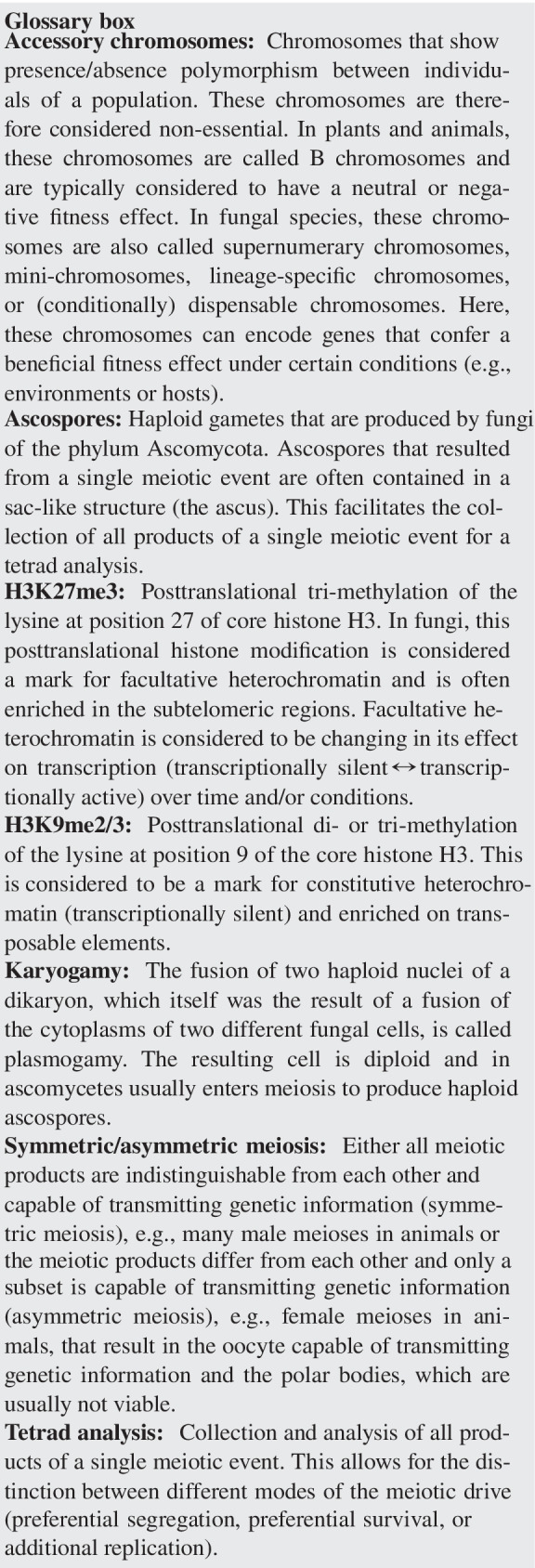


Meiotic drives increase the frequency of a genetic element relative to the rest of an individual’s genome. Small transmission advantages due to a meiotic drive can result in a rapid invasion of the genetic elements despite a neutral or negative fitness effect. Genetic elements that increase their transmission by a meiotic drive are therefore often considered selfish genetic elements (Werren et al. 1988; Hurst et al. 1992; Lyttle 1993; Hurst et al. 1996; Hurst and Werren 2001; McLaughlin and Malik 2017; Gardner and Ubeda 2017; Ågren and Clark 2018; Courret et al. 2019). Several studies have demonstrated that the symmetry of the meiotic cell divisions can predispose a system to the invasion of a specific type of meiotic drives (Kruger and Mueller 2021). Meiotic as well as mitotic cell divisions can be categorized as being either symmetric, i.e., all daughter cells are indistinguishable from each other or asymmetric where the daughter cells differ from each other. Symmetric meiotic cell divisions, e.g., many male meiotic cell divisions in animals, appear to be more prone to the invasion of drives that cause a difference in the fitness of gametes with and without the drive element (Kruger and Mueller 2021). One well-described example is the segregation distorter (SD) in *Drosophila* affecting the male meiosis, where the sperm cells that do not carry the drive element become functionally defective (Hurst and Werren 2001; Courret et al. 2019). Similarly, in male mice, the t-haplotype represents a poison–antidote system that results in sperm mobility impairment (Herrmann et al. 1999, Bauer et al. 2005). In fungi, with symmetric meiosis, meiotic drive systems employing similar mechanisms seem to be prevalent. For example, in several species of the genus *Neurospora*, only the meiotic progeny (called ascospores) that carry the spore killer allele (*Sk-2* or *Sk-3*) survive, while ascospores without this allele typically deteriorate and die (Hammond et al. 2012; Svedberg et al. 2021). An additional example of an symmetric meiotic drive is the *wtf* poison–antidote system in yeast, where the *wtf* driver in *Schizosaccharomyces pombe* and *S. cambucha* encodes pre-meiotically expressed poison and the post-meiotically expressed antidote (Nuckolls et al. 2017; Hu et al. 2017), thus allowing only survival of spores carrying the *wtf* drive element. Similar systems are the *Het-s* drive and the *Spok* genes in the fungus *Podospora anserina* (Vogan et al. 2019; Grognet et al. 2014), as well as the qHMS7 locus in rice, *Oryza sativa* (Yu et al. 2018).

Asymmetric meiotic cell divisions, in which only a subset of the resulting meiotic daughter cells will provide genetic material to the next generation, on the other hand, appear to be more prone to the invasion of drive mechanisms that manipulate their segregation during the cell division. For instance, during female animal meiosis, asymmetric cytokinesis results in the production of the egg cell as well as polar bodies (which in many organisms usually die). Only the genetic material of the egg cell will be transmitted to the next generation. This asymmetric cell division is exploited by genetic elements that increase their segregation to the egg cell instead of to the polar bodies (Hurst and Werren 2001). In this regard, centromeres appear pivotal to the drive mechanism by affecting the segregation of the linked chromosomes; accordingly, these drive mechanisms are termed centromere drive (Henikoff et al. 2001; Malik 2009; Akera et al. 2017). Such a centromere drive has also been reported in monkeyflower (*Mimulus guttatus*) populations (Fishman and Willis 2005; Fishman and Saunders 2008) and in mice (Chmátal et al. 2014). In summary, based on examples from different groups of organisms, it is currently considered that the symmetry of meiotic cell divisions is one of the main factors that predisposes a system to the invasion of certain meiotic drive systems with either killing or segregation manipulation.

Accessory chromosomes are non-essential genetic elements in plants, animals, and fungi, and are often transmitted in a non-Mendelian way. These chromosomes, also known as B, supernumerary or (conditionally) dispensable chromosomes, are entire chromosomes that are by definition present in some but not all members of a population. Several fungal species (so far mainly described in genomes of plant pathogenic species) contain one or more distinct accessory chromosomes, which can encompass a sizable portion of the fungal genome (Galazka and Freitag 2014; Mehrabi et al. 2017; Bertazzoni et al. 2018; Habig and Stukenbrock 2020). Accessory chromosomes in fungi, in contrast to accessory chromosomes (called B chromosomes) in plants and animals, more often confer a fitness benefit for the host individual (Han et al. 2001) but with some examples where the accessory chromosomes confer a negative fitness effect (Balesdent et al. 2013; Habig et al. 2017; Rouxel and Balesdent 2017). Some of these fungal accessory chromosomes contain genes that are indeed essential for the successful infection of certain plant hosts (Hatta et al. 2002; Ma et al. 2010). Fungal accessory chromosomes are similar to B chromosomes in plants and animals in their deviation from Mendelian segregation during meiosis. This non-Mendelian inheritance of fungal accessory chromosomes appears to involve either chromosome losses and duplications or transmission of an accessory chromosome to more progeny than predicted by Mendelian segregation, i.e., meiotic drive (Coleman et al. 2009; Croll et al. 2013; He et al. 1998; Mehrabi et al. 2017; Orbach et al. 1996; Wittenberg et al. 2009; Xu and Leslie 1996; Fouché et al. 2018; Habig et al. 2018). Fungal accessory chromosomes are unique among accessory chromosomes due to their functional diversity and their differences in mechanisms causing non-Mendelian segregation and meiotic drive. In this review, we will focus on mechanisms that could explain non-Mendelian transmission and the meiotic drive of fungal accessory chromosomes. First, we will give an overview of the structural and functional features of accessory chromosomes, which may be affecting their deviating transmission pattern.

## Structure and function of accessory chromosomes in fungi

B chromosomes in plants and animals have been known since the beginning of the twentieth century (Wilson 1907). In contrast, the small accessory chromosomes of fungi were discovered much later by electrophoretic separation of chromosomes of different fungal individuals using pulsed-field gel electrophoresis (PFGE). Hence, the first reports of fungal accessory chromosomes in the phytopathogenic fungi *Nectria haematococca*, *Cochliobolus heterostrophus*, *Magnaporthe grisea*, and *Fusarium solani* date back only to the early 1990s (Miao et al. 1991; Mills and McCluskey 1990; Orbach et al. 1996; Talbot et al. 1993). Next-generation sequencing subsequently enabled more detailed analyses of accessory chromosomes in different fungal species. The comparatively small size of fungal genomes and the correspondingly short chromosome lengths facilitate these sequencing approaches. For many fungal organisms, chromosome-scale assemblies from telomere to telomere have been available for many years, including detailed annotations of genetic and epigenetic features. Furthermore, many fungi can easily be propagated in a haploid form, which facilitate sequencing and simplifies genomic analysis. Hence, fungi represent excellent model organisms for studying meiotic drive and chromosomes transmission mechanisms.

Fungal accessory chromosomes appear to share certain specific, albeit non-unique, sequence characteristics. Accessory chromosomes vary in size from ~ 0.2 to ~ 3.5 Mb (Habig and Stukenbrock 2020), are enriched in transposons or repetitive sequences, and show lower gene densities and differences in codon usage compared to the essential core (also called A) chromosomes (Coleman et al. 2009; Ma et al. 2010; Goodwin et al. 2011). The accessory chromosomes appear mostly heterochromatic with an enrichment of the histone modification H3K9me2/3 (see glossary box), which is associated with constitutive heterochromatin (Freitag 2017; Studt et al. 2016; Connolly et al. 2013; Schotanus et al. 2015). Interestingly, fungal accessory chromosomes appear to have a unique histone modification pattern by being also enriched with H3K27me3 throughout their entire length (Galazka and Freitag 2014; Schotanus et al. 2015; Connolly et al. 2013; Studt et al. 2016; Fokkens et al. 2018; Erlendson et al. 2017). Interestingly, H3K27me3 seems to be also involved in the unique pattern of histone modifications found on B chromosomes of rye (Carchilan et al. 2007; Gonzalez-Sanchez et al. 2014). The presence of facultative heterochromatin therefore appears to be a distinctive trait of fungal accessory chromosomes while showing some overlap with epigenetics marks of B chromosomes. Finally, it is important to note that accessory chromosomes of fungi cannot be identified exclusively based on their sequence composition and structural characteristics. In some cases, core chromosomes can also comprise segments of low gene density, distinct heterochromatin structures, and high repeat content. Therefore, identifying accessory chromosomes in fungi currently solely relies on their presence/absence polymorphism.

Several fungal accessory chromosomes appear to have a positive fitness effect—at least under certain conditions. The functional relevance of fungal accessory chromosomes has mainly been addressed in plant pathogenic species (Bertazzoni et al. 2018; Mehrabi et al. 2017; Soyer et al. 2018; Habig and Stukenbrock 2020). Some of these chromosomes contain genes required for pathogenicity or growth within specific plant hosts or conditions. As an example, in the filamentous fungus *Alternaria alternata*, the AK-toxin gene cluster controlling host-specific pathogenicity resides on a 1.05-Mb accessory chromosome and the absence of this chromosome leads to avirulent phenotypes of this plant pathogen (Hatta et al. 2002). Likewise, the accessory chromosome 14 of the plant pathogenic isolates of *Fusarium oxysporum f.* sp. *lycopersici* contains genes required to infect tomatoes (Ma et al. 2010). It is important to note, that in a few cases, fungal accessory chromosomes do confer a fitness disadvantage. In the wheat pathogen *Zymoseptoria tritici*, the deletion of whole accessory chromosomes resulted in strains with increased fitness (Habig et al. 2017). Hence, the fitness effects of fungal accessory chromosomes appear to be—in contrast to the B chromosomes in plants and animals—only in a few cases negative but mostly neutral or positive. Based on the identification of virulence-related genes on accessory chromosomes, it has been suggested that these small chromosomes represent dynamic genomic compartments promoting the rapid evolution of new adaptive traits (Croll and McDonald 2012). Such compartments with increased rates of evolution may be favored by the co-evolutionary interaction of pathogens with their host without affecting conserved housekeeping genes encoded by core chromosomes (Chuma et al. 2011; Taylor et al. 2017). In support of such a scenario, we recently demonstrated that the mutation rate on accessory chromosomes indeed is significantly higher than on the core chromosomes (Habig et al. 2021).

The origin of fungal accessory chromosomes is still unresolved and includes two main non-exclusive hypotheses. The first speculates that accessory chromosomes originated from core chromosomes (Galazka and Freitag 2014). This is supported by the distribution of families of repetitive elements (Grandaubert et al. 2015) that are shared between core and accessory chromosomes. Furthermore, genome sequencing of meiotic progenies demonstrated a breakage-fusion-bridge cycle as a process that gave rise to a new accessory chromosome in *Z. tritici* (Croll et al. 2013). In addition, recent macrosynteny analyses in the rice blast pathogen *Magnaporthe oryzae* followed the emergence of accessory mini-chromosomes via structural rearrangements and segmental duplication of core chromosomes (Langner et al. 2021). The second hypothesis of accessory chromosome origin proposes that accessory chromosomes originate via horizontal chromosome transfer from different lineages or species (Mehrabi et al. 2011). This idea is supported by differences in codon usage between core and accessory chromosomes (Goodwin et al. 2011) as well as experimental or phylogenetic evidence of horizontal transfer of entire chromosomes between distinct lineages (Akagi et al. 2009a, b; He et al. 1998; Ma et al. 2010; Masel et al. 1996). Since accessory chromosomes might have a different inheritance pattern (see next section), it is important to note that differences in sequence composition of core and accessory chromosomes may not necessarily relate to distinct origins but rather reflect differences in mutational processes, epigenetic marks, transmission, and evolutionary histories.

## Non-Mendelian segregation and meiotic drives of accessory chromosomes in fungi

**Fig. 1:**
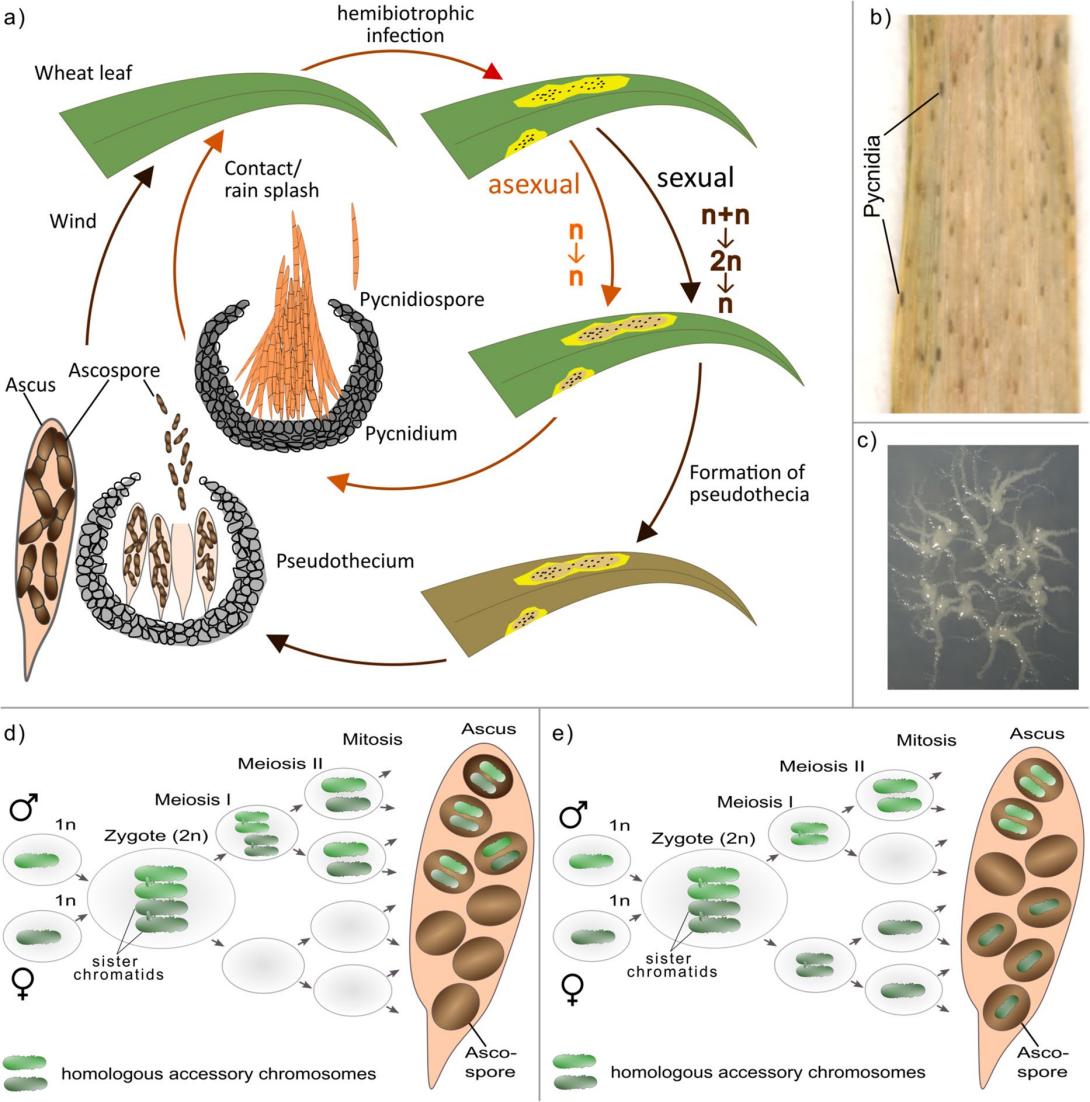
**a** Life cycle of *Z. tritici* as an example of an Ascomycete life cycle containing both asexual and sexual reproduction. Asexual reproduction results in pycnidiospores (asexual haploid spores), produced by asexual fruiting body (pycnidia) in infected wheat leaves (adapted from Ponomarenko 2011). The asexual cycle is assumed to only contain haploid cells. Sexual reproduction in *Z. tritici* results in ascospores (sexual haploid spores) produced in the sexual fruiting body (pseudothecium). Here, two specialized haploid cells fuse to form a diploid zygote which undergoes two meiotic cell divisions and one mitotic cell division to produce eight haploid ascospores contained in a sac-like structure (ascus). **b** Example pictures of asexual fruiting body (pycnidia) formed by *Z. tritici* on necrotic leaf tissue lesion carrying asexual pycnidiospores on a wheat leaf infected with *Z. tritici*. **c** Example picture of in vitro growth of *Z. tritici* growing on xylose minimum medium. **d–e** Models for losses and disomies of fungal accessory chromosomes due to nondisjunction of **d** homologous chromosomes or **e** sister chromatids during meiotic cell divisions. **d** Homologous accessory chromosomes fail to segregate at first meiotic division which leads to their disomy in half of the progeny and the absence of respective homologous accessory chromosomes in the remaining half of ascospores. **e** Nondisjunction of sister chromatids during the second meiotic division causes loss and disomy of accessory chromosomes. In contrast to disomic progeny from **d**, disomic accessory chromosomes are identical and chromosomes are absent in 25% of the progeny. For the sake of clarity, no recombination events are depicted

Many fungi have a sexual reproductive cycle but can also reproduce asexually (see Fig. 1). In addition, the distinction between somatic and germline cells cannot be defined in fungi, since individual cells are—in most cases—totipotent. Hence, the distribution of accessory chromosomes is not solely affected by their inheritance during meiosis but also by their transmission during mitotic growth. We will therefore use the term “transmission” to generally describe the behavior of accessory chromosomes during cell divisions, considering both mitotic and meiotic cell divisions, to emphasize that both types of cell divisions affect the presence/absence polymorphisms of accessory chromosomes. We will briefly introduce transmission patterns of fungal accessory chromosomes during mitotic divisions before focusing on their transmission during meiotic cell divisions. It is important to note, that the small size of fungal accessory chromosomes makes the microscopic analysis of their transmission during mitotic or meiotic cell divisions very challenging. At the same time, this small chromosome size in concert with the ease of access to haploid cells and, in the case of sexually reproducing fungi, the ease of access to entire tetrads allows a very detailed analysis of their transmission pattern via whole-genome sequencing. The availability of high-quality genome assemblies spanning chromosomes from telomere to telomere further facilitates such genome-based analyses, which are therefore the focus of this review.

Fungal accessory chromosomes are frequently lost during mitotic propagation in several fungi. In the asexual tomato pathogen, *F. oxysporum f.* sp. *lycopersici* accessory chromosomes are lost during mitotic growth at a frequency of 1 in 35,000 spores (Vlaardingerbroek et al. 2016). Much higher loss rates of accessory chromosomes have been reported for members of the genus *Zymoseptoria*. Here, *Z. tritici* and its sister species *Z. ardabiliae* showed a loss of accessory chromosomes in 1 out of 50 spores (Möller et al. 2018; Habig et al. 2021). Interestingly, temperature greatly impacts the stability of chromosomes whereby an increased temperature (28°C) dramatically increases the loss rate of accessory chromosomes in *Z. tritici* (Möller et al. 2018; Habig et al. 2021). These increased losses of accessory chromosomes during mitotic cell divisions do not appear to be a result of sister chromatid nondisjunction during mitosis but rather due to aberrant DNA replication possibly impacted by the histone modification H3K27me3 (Habig et al. 2021). Accessory chromosomes in *Z. tritici*, *F. oxysporum*, *F. asiaticum*, and *Gibberella fujikuroi* are enriched in the histone modification H3K27me3 (Galazka and Freitag 2014; Schotanus et al. 2015; Fokkens et al. 2018). This histone modification appears to destabilize accessory chromosomes and increase the mutation rate (Möller et al. 2019; Habig et al. 2021). H3K27me3 appears to be a hallmark of accessory chromosomes as H3K27me3 on core chromosomes is restricted to the subtelomeric regions and moreover associates these regions with the nuclear envelope (Erlendson et al. 2017; Harr et al. 2015, 2016). We speculate that the enrichment throughout the entire length of the fungal accessory chromosomes might associate the entire chromosome with the nuclear envelope and thereby affect DNA replication (Möller et al. 2019; Habig et al. 2021) and the transmission of accessory chromosomes during mitotic divisions. Possibly, this effect of histone modifications on DNA replication of accessory chromosomes could also be pivotal during the meiotic transmission of the accessory chromosomes and their meiotic drive.

Accessory chromosomes often show a non-Mendelian transmission during meiotic cell divisions in fungi that undergo sexual reproduction. This can involve either chromosome losses or segregation distortion and/or meiotic drives. Losses of accessory chromosomes during meiotic cell divisions appear frequently. As an example, *L. maculans*, the stem canker agent of oilseed rape (*Brassica napus*), contains an accessory chromosome (termed conditionally dispensable chromosome (CDC) in this species) that is lost in approximately 5% of the progeny following meiosis (Balesdent et al. 2013; Leclair et al. 1996). It is however unclear whether this loss is accompanied by disomy of the same chromosome in one of the other products of the meiotic cell division—and therefore is the result of nondisjunction of chromosome homologs or sister chromatids. Nondisjunction events that lead to the loss of accessory chromosome occur during first or second meiotic divisions and are associated with a corresponding chromosome duplication or disomy (Fig. 1). The accessory chromosome of the rice blast fungus *M. oryzae* (called mini-chromosomes in this species) also shows non-Mendelian transmission during crosses due to nondisjunction during meiosis I or meiosis II (Orbach et al. 1996). Loss and corresponding disomy of the accessory chromosome PDA1-CDC in *N. haematococca* MPVI (called conditionally dispensable chromosome in this species) suggest the occurrence of nondisjunction of chromosomes in meiosis I (Miao et al. 1991; Garmaroodi and Taga 2007, 2015). Accessory chromosomes in *Z. tritici* also show non-Mendelian inheritance with up to 20% of the progeny from a meiotic cross being reported to have lost one or more accessory chromosomes (Fouché et al. 2018; Wittenberg et al. 2009). Correspondingly, disomies of accessory chromosomes are a frequent result of meiosis in *Z. tritici*, which would indicate meiotic nondisjunction events (Fouché et al. 2018). Furthermore, we confirmed both nondisjunctions as well as losses of accessory chromosomes in *Z. tritici* via tetrad analysis of all meiotic products of individual meioses and showed that simultaneously nondisjunctions were absent for the core chromosomes (Habig et al. 2018). In summary, non-Mendelian inheritance due to nondisjunction appears to be a common process for fungal accessory chromosomes. One could speculate that the apparent higher frequency of nondisjunctions in fungal accessory chromosomes compared to core chromosomes could be due to either (i) a functional difference in the centromeres of the accessory chromosomes or (ii) a higher probability to observe nondisjunctions in accessory chromosomes due to their non-essential role and therefore possibly lack of lethal gene-doses effect or (iii) mechanistic differences in the meiosis of core and accessory chromosomes. In *Z. tritici*, the centromeres of core and accessory chromosomes are indistinguishable based on their sequence composition and structure (Schotanus et al. 2015). Nevertheless, disomies of certain core chromosomes occur during longtime mitotic propagation ruling out a lethal gene-doses effect of these disomies. Consequently, it is very plausible that the difference in the frequency of nondisjunctions for core and accessory chromosomes relates to differences in their meiotic behavior. Nondisjunction events will not by themselves change the relative frequency of accessory chromosomes in a population compared to core chromosomes, provided that disomies of accessory chromosomes have no fitness effect. Therefore, other mechanisms conferring a transmission advantage or a fitness advantage are likely involved in the maintenance and the dynamics of accessory chromosomes in fungal organisms.

Several fungal accessory chromosomes increase in frequency during meiotic cell divisions (Balesdent et al. 2013; Fouché et al. 2018; Goodwin et al. 2011; Tzeng et al. 1992; Wittenberg et al. 2009). In fungi of the phylum *Ascomycota*, haploid individuals can sexually reproduce by fusion of specialized cells (plasmogamy) followed by fusion of the two separate haploid nuclei (karyogamy) eventually resulting in a diploid zygote which then undergoes meiosis to produce the haploid meiotic products. These meiotic products are contained in an eponymous sac (ascus) (see Fig. 1). By detailed analyses of the ascospores contained in one ascus, the transmission of the accessory chromosomes from the haploid parental strain via the diploid zygote to the haploid ascospores can be easily tracked. The non-essential nature of accessory chromosomes allows for a more encompassing analysis of their transmission as any loss, disomy, or other large-scale chromosomal events are non-lethal and therefore visible in the ascospore progenies. Although accessory chromosomes provide excellent model systems to study molecular mechanisms associated with meiosis, we still lack detailed insights into the underlying aspects of their transmission advantages. The first report of a transmission advantage of a fungal accessory chromosome was on accessory chromosome 16 of *C. heterostrophus*, the causal pathogen of the disease southern corn leaf blight (Tzeng et al. 1992). Although only one of the two haploid parental strains contained the accessory chromosome 16, two-thirds of meiotic products inherited the chromosome (Tzeng et al. 1992). Similarly, in *Leptosphaeria maculans*, the causal pathogen of stem canker of oilseed rape, 83% of the meiotic products contained the accessory chromosome (called mini-chromosome in this species), although only one of the parental strains contained the accessory chromosome (Balesdent et al. 2013). These transmission advantages of unpaired accessory chromosomes (i.e., chromosomes inherited from only one of the parental strains) may reflect the presence of a meiotic chromosome drive. Possibly this transmission advantage could be due to increased survival of meiotic progeny containing the accessory chromosome or alternatively the unpaired accessory chromosomes could be subject to an additional replication. Thus, the mechanism affecting the transmission of fungal accessory chromosomes in these two species still remains unclear. Tetrad analyses and genome analyses of ascospore progenies should be applied to these two species to identify the underlying mechanisms of accessory chromosome dynamics.

**Fig. 2:**
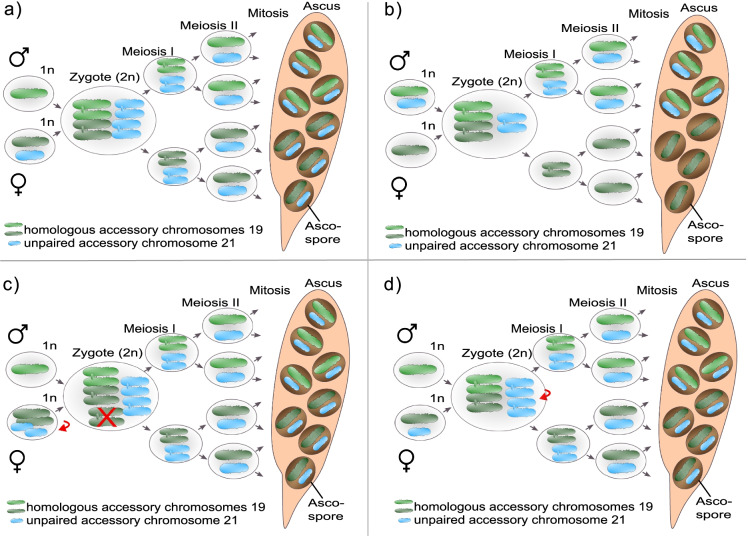
Meiotic drive of accessory chromosomes in *Z. tritici*. **a** Accessory chromosomes show presence/absence polymorphisms among individuals in populations and thus can be unpaired during meiosis. The chromosomal drive during meiosis in *Z. tritici* is restricted to female-inherited and unpaired accessory chromosomes (light blue) and causes an overrepresentation of female-inherited unpaired accessory chromosomes in the progeny. **b** Male-inherited unpaired accessory chromosomes (light blue) show Mendelian segregation as well as the paired accessory chromosomes (light green and dark green). The potential mechanism of the meiotic drive of accessory chromosomes likely involves additional replication that can happen either **c** prior to the fusion of two haploid gametes or **d** in the diploid zygote. If **c** is true, all chromosomes in the female haploid gamete should be amplified (red arrow), and in the zygote, the additional copies of paired accessory chromosomes must be deleted (red cross). Alternatively, **d** the unpaired female-inherited accessory chromosomes are amplified in the zygote. For simplicity, recombination events are not shown

Tetrad analysis—the collection and analysis of all products of a single meiosis—can distinguish pre- and post-meiotic effects and has been conducted for the accessory chromosomes in the wheat pathogen *Z. tritici*. Here, unpaired accessory chromosomes are transmitted to all haploid meiotic progenies (called ascospores) revealing the presence of a meiotic drive mechanism (Habig et al. 2018). Interestingly, this meiotic drive affects only those unpaired accessory chromosomes of *Z. tritici* that were inherited from the parental strain that also provided the mitochondria to the ascospore progenies (i.e., the female parent) (Habig et al. 2018) (see Fig. 2). In contrast, when the same unpaired accessory chromosome from the same parental strain was inherited from the male parent (i.e., not providing the mitochondria), the accessory chromosomes followed Mendelian segregation and were transmitted to ~ 50% of the progeny (see Fig. 2). Interestingly, accessory chromosomes that had a homolog in each of the two parental strains also showed Mendelian segregation as well as recombination, implying canonical homologous pairing and segregation (Habig et al. 2018). This meiotic drive therefore appears mechanistically different from all previously characterized meiotic chromosome drives.

To date, meiotic drives have been mostly categorized into two main mechanisms: (i) preferential segregation during asymmetric pre-meiotic, meiotic, or post-meiotic cell divisions or (ii) disruption of gametes that do not carry the drive element (Hurst and Werren 2001; Werren 2011). Both of these mechanistic categories do not include an additional amplification of the drive element in sensu stricto. The fact that paired accessory chromosomes of *Z. tritici* follow Mendelian segregation while the same chromosomes show a meiotic drive when being unpaired and inherited from the female parent could be explained by either (i) an additional replication of all accessory chromosomes from the female parent followed by a selective elimination of all additional copies of paired accessory chromosomes (Fig. 2) or (ii) an additional amplification of unpaired accessory chromosomes in the zygote (see Fig. 2). It is important to note that complete tetrads could be isolated, and hence, no spore death is associated with the drive. Neither preferential segregation nor disruption of gametes would allow for the isolation of complete tetrads; therefore, these mechanisms cannot be responsible for the observed meiotic drive of female-inherited unpaired chromosomes (Habig et al. 2018). A recent tetrad analyses in *Z. tritici* could further pinpoint an additional replication of accessory chromosomes prior to zygote formation (unpublished data, Fig. 2). Consequently, the meiotic drive in *Z. tritici* relies on a preferential elimination of additional copies of the accessory chromosomes that do have a homolog in both strains. The exact mechanism for this drive is however still unclear.

One of the main open questions is how core and accessory chromosomes are distinguished by the cell. We speculate that particular epigenetic marks could be responsible, in particular, H3K27me3, which appears to distinguish accessory chromosomes from core chromosomes (Schotanus et al. 2015), possibly via its effect on the localization of these chromosomes within the nucleus (Möller et al. 2019) and/or their replication (Habig et al. 2021). Recently, we could show that H3K27me3 affects the replication of accessory chromosomes in *Z. tritici* during mitotic propagation (Habig et al. 2021), which indicates that their replication might also be affected by H3K27me3 during meiotic transmission, possibly also playing a role in their meiotic drive. We speculate that H3K27me3 might associate the accessory chromosomes with the nuclear envelope (Möller et al. 2018; Habig et al. 2021) and that this difference in location compared to the core chromosomes can contribute to the additional replication and meiotic drive. We are currently testing this hypothesis. In summary, our crossing experiments provide evidence for the relevance of a meiotic drive of accessory chromosomes in *Z. tritici* ensuring their maintenance over evolutionary time despite their fitness cost (Habig et al. 2017). Indeed, a similar drive mechanism could exist for other fungal accessory chromosomes for which a transmission advantage is observed.

## Concluding remarks

Although non-Mendelian segregation and meiotic drive appear frequent for fungal accessory chromosomes, they are mechanistically much less understood than those affecting B chromosomes in plants and animals or other genetic drive mechanisms. With fungal accessory chromosomes mainly described for pathogenic fungi, and containing genes affecting the pathogenicity, we argue that their transmission during meiosis should warrant detailed analysis. The identification of a potentially novel mechanism for a meiotic drive in *Z. tritici* serves as an example encouraging the potential discoveries in other fungal species. The general question, which unique features of accessory chromosomes affect or regulate their transmission during meiosis, is however not restricted solely to fungal accessory chromosomes but to all chromosome affected by a meiotic drive. We speculate that epigenetic modifications could be—at least for the fungal accessory chromosomes—such a distinguishing characteristic. These epigenetic modifications could play a role in the non-Mendelian transmission of accessory chromosomes in fungi but also could potentially be involved in the unique transmission pattern of B chromosomes in other eukaryotes. The small genome sizes, the general ease of access to haploid parental strains and meiotic progeny, and the non-essentiality of fungal accessory chromosomes will further facilitate studies on general meiotic processes. We consider fungal accessory chromosomes as a remarkable class of genomic components that can provide novel insights into mechanisms regulating the meiotic and mitotic transmission of chromosomes in general as well as the atypical inheritance of B chromosomes in other eukaryotes.
